# Mixed methods assessment of women’s risk of intimate partner violence in Nepal

**DOI:** 10.1186/s12905-019-0715-4

**Published:** 2019-01-28

**Authors:** Cari Jo Clark, Gemma Ferguson, Binita Shrestha, Prabin Nanicha Shrestha, Brian Batayeh, Irina Bergenfeld, Stella Chang, Susi McGhee

**Affiliations:** 1 Hubert Department of Global Health, Rollins School of Public Health, Emory University, 1518 Clifton Road, NE, Atlanta, GA 30322 USA; 2 Equal Access International, 1212 Market Street, Suite 200, San Francisco, CA 94102 USA

**Keywords:** Intimate partner violence, Social norms, Nepal, Couples, Mixed-methods

## Abstract

**Background:**

Intimate partner violence (IPV) is a significant public health issue that affects one in three women globally and a similarly large number of women in Nepal. Although important policy and programmatic steps have been taken to address violence against women in Nepal over the past decade, there is still a gap on IPV research in Nepal, particularly with regard to social norms.

**Methods:**

This mixed-methods study used in-depth interviews with women and their husbands as well as baseline survey data from a cluster randomized trial testing a primary prevention intervention for IPV to examine the prevalence and risk factors for IPV. Baseline survey data included 1800 women from Nawalparasi, Chitwan, and Kapilvastu districts in Nepal. Multivariate regression was used to identify risk and protective factors for exposure to physical and / or sexual IPV in the prior 12 months. Case-based analysis was used to analyze one of 18 pairs of in-depth interviews to examine risk and protective factors within marriages.

**Results:**

Of 1800 eligible participants, 455 (25.28%) were exposed to IPV. In multivariate analyses, low caste, wife employment, income stress, poor marital communication, quarrelling, husband drunkenness, exposure to IPV as a child, in-law violence, and gender inequitable normative expectations were associated with IPV. The selected case interview represented common themes identified in the analysis including the wife’s exposure to violence as a child, husband alcohol use, and marital quarrelling.

**Conclusions:**

Gender inequitable norms in the community and the intergenerational transmission of attitudes and behaviors supportive of IPV are important to address in intervention measures.

## Introduction

Intimate partner violence (IPV) is a serious public health issue and a violation of human rights, affecting one in three women globally [1]. IPV has well-documented adverse effects on women’s health [2–11] and substantial micro- and macro-economic implications [12–14]. In light of the health, social, and economic costs of IPV, there have been calls for better data on its prevalence, causes, and consequences to improve interventions aimed at addressing of violence against women and girls (VAWG) [15]. This global pressure led to the Sustainable Development Goal (SDG) 5.2, which calls for the elimination of all forms of VAWG in both public and private spaces (e.g., trafficking, sexual, and other types of exploitation) [16]. This manuscript is responsive to this call by examining the prevalence of IPV and its individual, couple, familial, and contextual risks and protective factors in Nepal.

## Background

### Prevalence of IPV in Nepal

IPV is a relatively new concept in Nepal and is understood as “*gharelu hinsha*” or “*mahila hinsa,*” phrases which refer to domestic violence and gender-based violence (GBV), respectively. These phrases infer other forms of IPV such as polygyny, extramarital affairs, beating, neglect, and verbal abuse [17]. IPV is widespread in Nepal; about one quarter (26%) of ever-married women have ever experienced physical, sexual, or emotional IPV, with the most common type being physical (23%) [18]. Fortunately, there has been a 6% decline of reported IPV in Nepal since 2011, due to declines in emotional and sexual violence [18].

### Framework of IPV in Nepal

The causes of IPV in Nepal are multifactorial [17, 19, 20]. In Nepal and elsewhere, low education [18], prior exposure to parental partner violence during childhood [17, 21, 22] and husband’s alcohol abuse [18, 23–26] have been shown to be associated with risk of IPV for women. Poor communication between spouses and frequent quarrelling are common correlates of IPV [27, 28]. Within Nepal and elsewhere where in-laws wield power over couple interactions, IPV often co-occurs with direct violence perpetrated against the daughter-in-law or by instigation of violence through their sons [17, 29]. Financial stress is often associated with IPV. It is a cause of stress and quarrelling which could erupt in situational violence [23, 30]. It may be used to reassert men’s authority in the household, which is undermined by joblessness and poverty [31], and lack of income may limit women’s options for recourse in abusive relationships [17, 32]. At the societal level, gender norms shape how men and women should act in a relationship [33]. In Nepal, as elsewhere, traditional gender norms reinforce aggression and dominance among men [34], increase acceptance of partner violence [34], and act as barriers to education and employment for women, increasing women’s risk of IPV [23, 26].

There are still gaps to understanding the range of risk and protective factors, especially those that also include perceptions of gender norms, which have not been explored in Nepal alongside more traditionally examined demographic and behavioral factors such as education and alcohol use. Further, prior risk and protective factors research in Nepal was either exclusively quantitative or qualitative. In this study, we contextualize risk and protective factors identified in survey data through analysis of in-depth interviews with couples in Nepal.

## Methods

### Overview

This analysis relies on data from the *Change Starts at Home* trial, which had the overall goal of reducing the occurrence of intimate partner violence through a social behavior change communication (SBCC) intervention which includes radio programming, listening and discussion groups (LDGs), and community outreach on physical and / or sexual IPV (NCT02942433) in three districts in Nepal (Nawalparasi, Chitwan, and Kapilvastu). The trial utilized a concurrent mixed-methods design [35] which has been described in detail elsewhere [35]. The present manuscript is a secondary data analysis of existing trial data. Relevant to this manuscript are data from a baseline survey of a community-based sample of reproductive age women and in-depth interviews with LDG participants which are described in detail below. The trial adheres to internationally recognized ethical standards for research on violence against women [36, 37]. All participants provided written informed consent.

### Sample

The *Change Starts at Home* project recruited 1800 married women in the Terai region of Nepal, selected for high IPV prevalence [18] and the existing presence of our implementing partner, Vijaya Development Resource Center [35]. Twelve village development committees (VDCs) in each district were purposively selected for practical reasons related to program implementation, demographics, and geographical separation [35]. VDCs were then pair-matched by the implementing partner according to demographic characteristics within each district, and two wards within each VDC were selected using probability proportionate to size methodology [35]. Simple random sampling was used to recruit 20 women per ward for a total community sample of 1440 women [35]. To be included in the sampling frame, participants had to be 18–49 years old, have a husband at least 18 years old with whom they resided for a majority of the year, and reside in the study area [35]. Ten couples from each intervention ward (*N* = 360) were purposively selected for the weekly LDG sessions with an emphasis on individuals who met the eligibility criteria, lived near the likely site of the LDG group, and were willing to commit to weekly participation for 9 months [35]. Six couples per district were selected for in-depth interviews from among the 360 couples participating in LDGs [35], and one of these couples was highlighted in the case study for this analysis.

### Measures

Surveys were developed in English from validated sources where available, translated into Nepali, and back-translated to ensure accuracy. Socio-demographic covariates assessed included district of residence, age at marriage in years (< 15, 15–17, 18–20, 21+), type of marriage (love marriage with and without parental blessing and arranged marriage with and without participant’s blessing), all modeled as categorical variables. The participants’ and their husbands’ educational levels (none, primary, some secondary, and School Leaving Certificate) were modeled as continuous variables given prior research showing a graded relationship [18]. Survey respondents were also asked if they had earned money for work or trade during the past 12 months and if so, if they made more, less, or the same amount as their husbands (modeled as a categorical variable, reference “unemployed”). Respondents reported if they or their husband frequently felt stressed because of not having enough income. Caste/ethnicity was categorized into upper caste and relatively advantaged Janajatis, disadvantaged non-Dalit and Janajatis, and Dalit and religious minorities as previous research in Nepal has found lower caste and religious minority status to be associated with a higher risk of IPV [23].

The frequency of communication between the respondent and her husband in the prior week (never, once, few, many times) was assessed with items from the World Health Organization’s Multi-Country Study on Health and Domestic Violence Against Women (WHO MCS) [32]. Topics assessed included “things that happened to him during the day”, “things that happened to you during the day,” “his worries or feelings,” and “your worries or feelings.” The score was calculated as a mean across the items. The frequency of quarrelling (never, sometimes, often) and husband’s inebriation (never, once a month or less, at least weekly) were assessed with one item each from the WHO MCS [32]. Both were modeled dichotomously denoting quarrelling at least sometimes (reference “never”) and being drunk at least weekly (reference “once a month or less” or “never”).

A measure of in-law violence was developed for this study based on research in South Asia, including Nepal, highlighting the role of in-laws in women’s risk of IPV [38] and prior research measuring in-law violence [29]. The participant was considered to have experienced violence by an in-law if she responded affirmatively to items assessing emotional (called names, insulted, humiliated or prevented from leaving the home) or physical (hit, kicked, punched or otherwise physically hurt) abuse, or reported that her husband’s family encouraged him to hit, kick, punch, or otherwise physically hurt her. Exposure to IPV as a child was assessed with a single item. Given the high percentage of “don’t know” responses regarding husband’s exposure, the husband’s exposure to IPV as a child was categorized as no, yes, and don’t know.

Gender equitable attitudes were measured with 10 items derived from the Gender-Equitable Men (GEM) scale, a 24-item scale with Cronbach’s alpha 0.81 [39]. A score was calculated as a sum across the items, with a higher score representing more gender equitable attitudes. The Partner Violence Norms Scale (PVNS) was developed for the study [33] to measure normative expectations with items measuring traditional gender role expectations (2 items), intra-familial dynamics (1 item), acceptability of violence (1 item), silence and tolerating violence to preserve the family and family honor (2 items), non-interference in family affairs (1 item), and appropriate expressions of women’s sexuality (1 item) [33]. The score was calculated as a sum across the eight items with higher scores representing more gender equitable norms in their community.

Physical and / or sexual IPV in prior 12 months was measured with the standard items employed through the What Works to Prevent Violence Global Program [35]. Items assessed the frequency of occurrence (never, once, few, many) of five items measuring physical IPV and three items measuring sexual IPV. Reported occurrence of any item in the prior 12 months constituted exposure to IPV. Cronbach’s alpha for the scale was 0.90. The measure was modeled dichotomously as exposure to any of the physical or sexual IPV experiences in past 12 months compared to no experience in the prior 12 months.

### In-depth interviews

Individual in-depth interviews with participants (*n* = 18) and their husbands (n = 18) within the LDG couple cohort were conducted by professionally trained facilitators at program launch. Separate interviews were conducted for husbands and wives. The semi-structured interviews consisted of 20 open-ended questions related to personal attitudes, beliefs and expectations as well as marital, familial and community dynamics. Examples of questions include, “As a wife, what do you expect from you husband?” “Do you share your opinion or inner feelings with your spouse? What do you share with him / her?” and “How do you and your spouse manage conflict?”. Each interview lasted approximately 45–90 min. With consent of participants, the interviews were recorded and thereafter transcribed and translated directly from Nepali into English.

### Data analysis

All quantitative analyses were completed using SAS 9.4. Multivariate logistic regression was used to assess the relationship between IPV and the proposed covariates outlined above. Generalized estimating equations were used to account for cluster sampling. For the in-depth interviews, case- and code-based analysis were used to examine risk and protective factors within marriages. Employing the existing codebook for the parent study, team members in the United States and Nepal coded a subsample of transcripts. Emergent codes were discussed by team members and incorporated into the final codebook, which was applied by team members in both countries. Preliminary case summaries of each couple were compiled and displayed using matrices to determine overall and code-specific change, considering reports from either spouse. To clearly portray these risk factors in context, one couple was selected based on the clarity and range of risk factors present. While the couple does not represent the entire qualitative cohort, the risk factor relationships described represent robust themes present throughout the dataset.

## Results

### Quantitative

Overall, 15.67% (*N* = 282) of the respondents reported physical violence, 18.07% (*N* = 325) reported sexual violence, and 25.28% (*N* = 455) reported physical and/or sexual violence. Table 1 shows distribution of covariates within the sample and their associations with physical and / or sexual IPV, using a fully adjusted model. When examined simultaneously in a multivariate logistic regression, caste **(**Disadvantaged non-Dalit and Janajatis, OR 1.71 [95%CI = 1.23, 2.38]) was associated with increased odds of being exposed to IPV. With regard to financial factors, multivariate analyses indicated that participant employment (less earnings than husband, OR 1.49 [95%CI = 1.08, 2.04]; equivalent earnings to husband’s earnings, OR 1.79 [95%CI = 1.28, 2.50]), and experiencing financial stress were associated with a higher likelihood of IPV exposure (OR 1.59, [95%CI = 1.22, 2.09]). Considering couple relations, we found that couples who quarreled were more likely to be exposed to IPV (OR 4.55, [95%CI = 3.26, 6.35]), and that husbands who were drunk frequently were more likely to perpetrate IPV (OR 2.38, [95%CI = 1.69, 3.36]). Taking the larger family into context, findings indicated that exposure to IPV as a child, for both wife and husband, was associated with increased likelihood of IPV exposure for the wife (OR 1.60 [95%CI = 1.19, 2.14]; OR 1.64, [95%CI = 1.17, 2.30], respectively), and that participants who experienced violence from their in-laws in past 12 months were more likely to have been exposed to IPV from their husbands (OR 2.82, [95%CI = 2.01, 3.96]). Additionally, when the respondent answered that they did not know whether the husband had ever been exposed to IPV as a child, they more likely to have been exposed to IPV within the past 12 months (OR 1.43, [95%CI = 1.06, 1.94]).

**Table 1:** Sociodemographic Characteristics of the Sample and Bivariate and Multivariate Association with Physical and / or Sexual IPV (*N* = 1800)

	Distribution		Multivariate	
	No.	%	OR	95%CI
Caste				
Uppercaste and relatively advantaged Janajatis	833	46.36	REF	
Disadvantaged non-Dalit and Janajatis	812	45.19	1.71**	**(1.23, 2.38)**
Dalit and religious minorities	152	8.46	1.46	(0.98, 2.18)
Age at Marriage				
< 15	226	12.56	REF	
15–17	646	35.89	0.92	(0.60, 1.41)
18–20	615	34.17	0.99	(0.65, 1.50)
21+	313	17.39	0.81	(0.49, 1.35)
Marriage Type				
Arranged w/consent	1148	63.78	REF	
Arranged w/o consent	170	9.44	1.32	(0.87, 2.01)
Love w/fam blessing	185	10.28	0.91	(0.57, 1.45)
Love w/o fam blessing	297	16.5	0.79	(0.56, 1.12)
Wife Education	1.31	1.10	1.13	(0.94, 1.34)
Husband Education	1.78	1.01	0.88	(0.76, 1.01)
Wife Employment				
Unemployed	941	52.28	REF	
Earns less than her husband	521	28.94	1.49*	**(1.08, 2.04)**
Earns the same amount as her husband	269	14.94	1.79**	**(1.28, 2.50)**
Earns more than her husband	69	3.83	1.44	(0.72, 2.87)
Income Stress	806	44.88	1.59**	**(1.22, 2.09)**
Marital Communication (mean/SD)	1.89	0.85	0.72**	**(0.62, 0.83)**
Quarrelling	1208	67.11	4.55**	**(3.26, 6.35)**
Husband Frequently Drunk	426	23.67	2.38**	**(1.69, 3.36)**
Wife Exposed to IPV As a Child	380	21.11	1.60**	**(1.19, 2.14)**
Husband Exposed to IPV As a Child				
No	1159	64.39	REF	
Yes	273	15.17	1.64**	**(1.17, 2.30)**
Don’t know	368	20.44	1.43*	**(1.06, 1.94)**
In-law Violence	184	10.26	2.82**	**(2.01, 3.96)**
Gender Equitable Attitudes (mean/SD)	1.10	0.49	0.99	(0.96, 1.02)
Gender Equitable Normative Expectations (mean/SD)	1.03	0.54	0.95**	**(0.92, 0.98)**
District				
Nawalparasi	600	33.33	REF	
Chitwan	600	33.33	0.66*	**(0.48, 0.91)**
Kapilvastu	600	33.33	0.98	(0.66, 1.44)

Values in bold indicate significant results: *P < 0.05; **P < 0.01

Alternatively, we found that couples with good communication had a lower risk of IPV (OR 0.72, [95%CI = 0.62, 0.83]). With regards to attitudes and norms, findings indicated that while gender equitable attitudes had no association with IPV exposure, participants who perceived their communities to be more gender equitable had a reduced likelihood of IPV exposure (OR 0.95, [95%CI = 0.92, 0.98]). Further, risk of IPV among women in Chitwan was lower than that of women in Nawalparasi in the fully adjusted model (OR 0.66, [95%CI = 0.48, 0.91]).

### Case study

Code and case-based analyses resulted in the development of case studies, which exemplify how risk and protective factors affect women’s risk of IPV. This case presented in this manuscript was selected as it represents common themes identified in the analysis, including the wife’s exposure to violence as a child, husband’s alcohol use, and marital quarrelling. While not included in the survey, but established by previous research, husband’s gender-equitable attitudes was also a common theme in the case study.

This particular couple had been married 7 years and shared two children. While it was unclear if the husband was exposed to abuse as a child, the wife explained, “*When I was small my dad would raise hands at my mother, and even my brother hitting 1-2 slaps*,” which may have normalized ideas of violence in marriage and influenced her tolerance of violence within her own marriage. Despite this, the wife expressed disapproval of both sexual and physical violence.

The husband expressed fairly gender equitable views related to sexual violence, “*Sexual relationship should be mutual. If both of us are not interested, then it shouldn’t be forced as well… forcing it on her is violence*”, and gendered division of labor, *“Others tell me that men don’t wash clothes, clean dishes and that it’s shameful washing or doing household chores. I don’t feel the same. I tell them - what is the shame in cleaning your own dishes or clothes that you bought with your own money*?.” His attitudes toward acceptability of physical violence, however, were less equitable, and posited that women’s reactions to violence against them were often dramatic, “*I have seen wives who leave their husbands after getting one or two slaps. They exaggerate, I see them getting one or two slaps. But they go around talking like as if they have been beaten up severely.”*

The presence of physical violence within their own marriage was discussed by both spouses, wherein they each situated violent incidents within quarrels induced by husband drunkenness. When asked about any bad habits of her husband, the wife described only one, “*No nothing at all. It’s just that he hits me when he is drunk”*. She explained that her husband became violent when she confronted and scolded him for being drunk: “*He gets angry, and I say you came home drinking, and he says don’t talk to me when I am drunk…you get hit so don’t speak a word, shut your mouth when am drunk”.* When asked the cause of the violence, the wife attributed it solely to her husband’s drunkenness and insisted that everything else remained positive: “*It’s only because of alcohol. There are no other reasons. Where ever I want to go, he does not object on that. If I want to go to have fun he never object, I don’t have money if you have you can go he says, or if you don’t have you can manage and go I’ll pay later”.*

Reflective of his attitudes about violence, the husband discussed incidents of physical violence lightheartedly, “*In those times, one or two slaps are given. (laughs) I say ‘How many times do I have to tell you not to talk to me when I am drunk’*”. During some of these incidents, he explained that his wife hit him back. He recalled his wife saying, “*If it’s okay for you to give me a slap, then I will slap you too* … *It’s not just you who has hands, I have too*”. He then further explained that there were limitations to these instances, “*When I am very angry, my wife doesn’t raise her hand*”. The wife explained she had begun trying to avoid violence when her husband was drunk: “*Now I won’t speak [about] how much he drinks or comes late at home…even if I want to, I won’t… I will keep my mouth shut*”. Due to this, the physical violence had decreased; however, intimidation and drunken threats of violence had persisted, which instilled fear in the wife, *“[He] does show me fear. I’ll hit you he says, and obviously I get scared by that”.*

Several factors, such as fairly frequent communication between the couple and the wife’s empowerment to leave the house whenever she wanted, may have been protective within this marriage. However, the interplay between wife’s exposure to violence, husband’s attitudes related to the acceptability of violence and alcohol-related quarrelling increased the risk for perpetration and victimization of IPV, including retaliatory violence on the part of the wife.

## Discussion

Approximately one quarter of women in our sample reported exposure to physical and / or sexual IPV in the past 12 months, which is approximately the same as the proportion of ever-married women reporting lifetime physical, sexual, and / or emotional violence in the 2016 Nepal DHS [18]. This suggests that lifetime estimates of IPV (including emotional IPV, which we did not measure) may be higher in our population, despite using similar items to assess IPV. This may be related to the fact that VDCs were selected by our implementing partner, so project activities could be occurring in areas with a demonstrated need for IPV interventions. It is also possible that women in the sample felt more comfortable disclosing IPV because of the established relationship with Vijaya Development Resource Center.

In line with current literature in Nepal and elsewhere, poverty and financial stress in our sample were significantly associated with past-year exposure to IPV [17, 19, 23, 40]. Also observed in prior research [18, 23–28], poor communication, alcohol abuse, quarrelling and childhood exposure to IPV were robustly associated with IPV risk, highlighting features of individual and relational behavior that are fundamental to the prevention of IPV. Sociodemographic characteristics, on the other hand, were among the least robust correlates of IPV in this study, despite being among the most studied correlates of IPV [41]. A notable exception was women’s employment, which was significant regardless of whether a wife earned less than or equal to her husband. The subsample of women who earned more than their husbands was too small to establish significance. Around the world, men are taught from an early age they are supposed to be the primary breadwinners in the family [30]. When men cannot provide for their families due to unemployment or poverty, they may use violence to reassert their position of power. For this same reason, caste-based systems are a contributory factor for risk of IPV as lower caste/ethnicity predisposes individuals to limited opportunities for socioeconomic advancement [42], which may potentially explain the lack of education’s significance in the fully adjusted model, as caste and socioeconomic status are interlinked. The problem is compounded for low caste women who face the double burden of gender and caste discrimination [43]. While several other sociodemographic factors were associated with IPV in bivariate models, most did not retain significance in a final model.

Finally, a measure of community gender norms was also a strong correlate of women’s risk of IPV, in alignment with a growing body of literature which is beginning to quantify this important relationship [33, 44, 45] and inform prevention interventions specifically targeting social norms. The link between individual attitudes and broader social norms is complex and worthy of further study. In this particular study, women’s individual attitudes toward gender equity and the acceptability of IPV were not robust correlates of women’s risk of IPV. However, in prior research in Nepal, measures of women’s attitudes have shown to be poorer predictors of IPV than men’s attitudes, [46, 47] which were not examined in this study.

The case profile exemplified and contextualized several risk and protective factors identified in previous research and through this study. Aligned with previous research, the wife’s exposure to IPV as a child may have increased her susceptibility to IPV [17, 21, 22], and although she expressed disapproval of IPV, violence in her own marriage was tolerated. Despite holding other gender equitable beliefs, the husband expressed blatant acceptability of VAWG, thus increasing risk for IPV perpetration [47]. Such attitudes reflect broader social norms related to acceptability of VAWG, which increases risk for women and girls in Nepal [17], and also highlights the potential disconnect between expressed beliefs and broader social context. While certainly not the sole cause of marital IPV, husband drunkenness contributed to marital quarrelling and husband aggression, aligning with the previously established association between alcohol use and IPV perpetration [17]. Based on these findings alone, one cannot argue causality with any certainty, but these findings largely reinforce existing research on the influence of IPV risk and protective factors, along the social ecological framework.

These results support ongoing efforts in Nepal to both better respond to and prevent IPV. In recent years, the Nepali government has taken numerous measure to improve laws related to VAWG and increase secondary and tertiary prevention for survivors [17]. For example, the 2010 National Action Plan Against Gender-Based Violence established an integrated approach to serving survivors and includes a focus on early detection, appropriate referrals and follow-up, and women and children’s legal empowerment [48]. Preliminary research identified several promising components of this initiative, including continuing support for survivors, accompaniment to legal system processes, and one stop crisis management centers [18, 49]. While these initiatives are a step in the right direction, the strength and enforcement of these laws and initiatives remains underdeveloped [50]. Further, these initiatives must be accompanied by stronger emphasis on the prevention of child maltreatment to stop the intergenerational transmission of abuse identified in this and many other prior studies, and need to more explicitly address the social norms that drive VAWG [17], which are inculcated early through the family [51] and reinforced throughout the life-course.

### Limitations

The study was fielded in only three districts in Nepal, suggesting that it may be too population-specific to be generalizable to Nepal as a whole. As has been demonstrated in other contexts, IPV is often underreported as women may fear retribution from husbands and in-laws or wish to avoid social embarrassment [17, 52]. Therefore, the prevalence estimate reported may underestimate the true prevalence of IPV. Finally, the study is cross-sectional and therefore temporality cannot be established and causality should not be inferred.

## Conclusion

This manuscript aimed to explore the main risk factors that affect the prevalence of IPV in Nepal. Through a mixed methods analysis, we identified a number of modifiable characteristics of individuals, relationships, and the broader society worthy of sustained action to prevent IPV. While the body of evidence on what works to prevent IPV is still growing, Nepal-based interventions are being tested [35] which if successful, will augment the prevention and response efforts already well-underway in the country.

## Abbreviations

CIs: Confidence intervals
GBV: Gender-based violence
GEM: Gender-Equitable Men Scale
IPV: Intimate partner violence
LDGs: Listening and discussion groups
ORs: Odds ratios
PVNS: Partner Violence Norms Scale
SBCC: Social behavior change communication
SDG: Sustainable Development Goal
VAWG: Violence against women and girls
